# A Study of the Wound Healing Mechanism of a Traditional Chinese Medicine, *Angelica sinensis*, Using a Proteomic Approach

**DOI:** 10.1155/2012/467531

**Published:** 2012-03-25

**Authors:** Chia-Yen Hsiao, Ching-Yi Hung, Tung-Hu Tsai, Kin-Fu Chak

**Affiliations:** ^1^School of Life Sciences, Institute of Biochemistry and Molecular Biology, National Yang-Ming University, Taipei 11221, Taiwan; ^2^Institute of Traditional Medicine, National Yang-Ming University, Taipei 11221, Taiwan; ^3^Department of Education and Research, Taipei City Hospital, Taipei 11221, Taiwan; ^4^Department of Medicine, Mackay Medical College, Taipei 25245, Taiwan

## Abstract

*Angelica sinensis* (AS) is a traditional Chinese herbal medicine that has been formulated clinically to treat various form of skin trauma and to help wound healing. However, the mechanism by which it works remains a mystery. In this study we have established a new platform to evaluate the pharmacological effects of total AS herbal extracts as well as its major active component, ferulic acid (FA), using proteomic and biochemical analysis. Cytotoxic and proliferation-promoting concentrations of AS ethanol extracts (AS extract) and FA were tested, and then the cell extracts were subject to 2D PAGE analysis. We found 51 differentially expressed protein spots, and these were identified by mass spectrometry. Furthermore, biomolecular assays, involving collagen secretion, migration, and ROS measurements, gave results that are consistent with the proteomic analysis. In this work, we have demonstrated a whole range of pharmacological effects associated with *Angelica sinensis* that might be beneficial when developing a wound healing pharmaceutical formulation for the herbal medicine.

## 1. Introduction

*Angelica sinensis* (AS), which is called *Dang-Gui* in Chinese, has been used in medicine for more than two thousand years in East Asia, including China, Japan, Korea, and India. In the past, AS has been mostly used to treat gynecological conditions and anemia [1, 2] or formulated with *Radix lithosperme* as an aid to wound healing [3]. In recent studies, AS has been shown to have multiple properties including the regulation of the immune system [4] and as antioxidant [5], antiinflammatory [6], anticancer [7] and others. Components of AS have been identified and classified into two groups; the essential oils and the water-soluble ingredients [8]. Ferulic acid (FA) is one of the most abundant water-soluble ingredients in AS and has been reported to be the active component of AS [9]. FA is prominent as a ROS scavenger because its structure is capable of stabilizing phenoxyl radical intermediates. Furthermore, FA is also able to activate proteins like heme oxygenase-1 (HO-1), heat shock protein 70 (HSP70), Erk *½*, and Akt, which help cells to respond to environmental stress [10, 11].

Herbal drugs are difficult to analyze due to their complexity and the presence of toxicity due to alkaloids. To avoid these complications, the effect of single active component of herbs is often explored, and this single active component represents the effects of the total herb; examples are shikonin for *Radix lithosperme* [12], and Oridonin for *Isodon rubescens* [13]. However, the disadvantage of this single component approach is that the results can never be the same as the complete biochemical and pharmacological mechanisms of the total herb and may not reveal the real mechanism(s) of the formulated traditional Chinese medicine.

Proteomics is a powerful tool and has been widely used to elucidate protein profile changes in response to drug treatment and to identify disease-relevant biomarkers. Using proteomics, *Rhizoma paridis* total saponin (RPTS) was identified as contributing to the anti-hepatocellular carcinoma effect (HCC) of this traditional medicine using HepG2 cells [14]. Similarly, a proteomic analysis on *Uncaria rhynchophylla* (Miq) Jack and its major component rhynchophylline was able to demonstrate an upregulation in the expression of MIF and cyclophilin A in kainic acid-induced epilepsy in rats [15].

*Angelica sinensis* (AS) is a basic component of many Chinese drugs that are used for wound healing, for example, shiunko [16]. Although AS has been applied in animal models and clinically, the mechanism by which AS helps wound healing remained to be clarified. Therefore the purpose of this study was to explore the mechanisms by which an ethanol extract of AS exerts its protective effect on human skin fibroblasts using both biochemical and proteomic approaches. This approach also explored the effect of the drug's active water-soluble component of FA. Based on these findings, it should be possible to identify the pharmacological effects of AS and how these contribute to the process of wound healing when treated with some Chinese traditional herbal medicines that contain AS.

## 2. Materials and Methods

### 2.1. HPLC Analysis

The HPLC system was equipped with BAS PM-80 pumps, a DGU-20A5 degasser, a CMA/170 autosampler, and a Varian (model 340) photodiode array detector. Chromatographic separation was performed using a Phenomenex Fusion RP-80 (2504.6 mm, 4 m). The mobile phases were acetonitrile (solvent A) and 2% acetic acid (solvent B). For the analysis of ferulic acid, the mobile phase involved the following linear gradient: from 25% A to 75% A in 0–15 min at a flow rate of 1.0 mL/min, and the detection wavelength was set on 280 nm. The sample injection volume was 20 *μ*L.

### 2.2. Cell Culture

Human embryonic skin fibroblast was obtained from Bioresource Collection and Research Center (BCRC) no. 60118 (Detroit 551). Cells were cultured in 10 cm culture dish (Corning) with minimum essential medium eagle (MEM) containing 2 mM L-glutamine, 1.5 g/L sodium bicarbonate, 0.1 mM nonessential amino acids, and 1.0 mM sodium pyruvate (MEM alpha-modifications, Sigma), supplemented with 10% fetal bovine serum (SAFC Bioscience), 50 units/mL penicillin, 0.05 mg/mL streptomycin, and 0.1 mg/mL neomycin (Sigma); the cells were grown in a humidified atmosphere at 37°C and 5% CO_2_. For passage, when fibroblasts had grown to a confluence of approximately 90% in the 10 cm culture dish, medium was discarded and the cells washed with 5 mL PBS twice. They were then harvested using 2 mg/mL EDTA and 5 mg/mL trypsin in phosphate-buffered saline (PBS). After washing with 5 mL PBS twice, the cells were collected by centrifugation at 1300 rpm for 5 min. The supernatant was discarded and the cells pipetted into fresh culture medium. They were then aliquoted into several 10 cm culture dishes.

### 2.3. Cell Viability Assay (WST-1 Assay)

The cell viability assay was performed according to the procedure described in the manufacturer's manual (Roche) with minor modifications. This is a colorimetric assay for the quantification of cell proliferation based on the cleavage of the water-soluble tetrazolium salt (WST-1) by mitochondrial dehydrogenases in viable cells. Drug-treated cells were seeded at a concentration of 4 × 10^4^ cells/well (about 40% confluence) in 1 mL culture medium into a 24-well culture plate and incubated at 37°C in an incubator containing 5% CO_2_. After cells had attached, they were treated with 0.1% DMSO, 300 *μ*g/mL AS extract, or 3.5 *μ*M FA for 24 hr. The supernatant was discarded and cell proliferation reagent WST-1 (Roche) was added to each well at a 1 : 50 ratio with fresh culture medium. The cells were then incubated for an additional 1 hr in dark. The absorbance was measured at 450 nm using an ELISA reader (Thermo, Multiskan Spectrum), and the absorbance of the background was measured at 690 nm. The difference in absorbance between 450 nm and 690 nm indicates the relative cell viability compared to 0.1% DMSO. The experiments were performed with three replicates for each sample.

### 2.4. Sample Preparation for 2D PAGE

Fibroblasts were washed twice in phosphate-buffered saline (PBS) and lysed with 1 mL of NP-40 lysis buffer containing 10 mM Tris-HCl (pH 7.5), 50 mM NaCl, 1% NP-40, 30 mM Na_2_P_2_O_7_, 30 mM NaF, 1 mM Na_3_VO_4_, 1% protease inhibitor, and 1% phosphatase inhibitor (Sigma). The whole cell lysate was centrifuged at 14000 rpm for 20 min at 4°C to remove insoluble material. The supernatant was transferred to a concentrator (GE Health, vivaspin 20, 100k MWCO) and centrifuged at 6000 ×g, 4°C, until volume less than 500 *μ*L, then the lysis buffer was replaced with dH_2_O containing 1% protease inhibitor and 1% phosphatase inhibitor (Sigma). The protein concentration was quantified by the Bradford protein assay (Bio-Rad), and the samples were applied directly for 2D PAGE analysis.

### 2.5. Analysis of the Protein Profiles Using 2D PAGE

The 2D PAGE was performed according to the method described in the manufacturer's manual (Amersham Biosciences) with minor modifications. For the first-dimension IEF, pH 4–7, IPG strips (18 cm) were rehydrated with 400 *μ*L rehydration buffer (0.5% IPG buffer, 8 M urea, 2% CHAPS, 50 mM dithiothreitol (DTT), and a trace of bromophenol blue) for at least 4 hr before 100 *μ*g of protein sample was loaded by cup loading. IEF was then carried out under following conditions: 150 V for 5 hr step and hold, 500 V for 3 hr step and hold, 1000 V for 7 hr gradient, 8000 V for 3 hr gradient and 8000 V for 18 hr step-and-hold. Prior to the second-dimension SDS-PAGE, the IPG strips were equilibrated with 4 mL of equilibration buffer, containing 50 mM Tris-HCl pH 8.8, 6 M urea, 2% SDS, 30% glycerol, 50 mM DTT, and 0.01% bromophenol blue at room temperature for 15 min, which was followed by equilibration in 50 mM Tris-HCl pH 8.8, 6 M urea, 2% SDS, 30% glycerol, 5% iodoacetamide, and 0.01% bromophenol blue at room temperature for 15 min. The second-dimensional SDS-PAGE used a 12.5% separating gel and was performed without a stacking gel. The equilibrated IPG gel strip was placed on top of the SDS-PAGE with appropriate pressure to ensure a firm contact between the strip and the gel slab. Electrophoresis was carried out at 35 mA/gel until the tracking dye reached the bottom of the gel. The 2D PAGE was then silver stained.

### 2.6. Fast Silver Staining

After 2D PAGE electrophoresis, the gel was removed, immersed in fix solution (40% methanol and 10% acetic acid in dH_2_O) for 10 min, washed with dH_2_O for 10 min twice, and then immersed in solution A (0.25 mM sodium thiosulfate) for 30 min, which was then replaced by dH_2_O for 10 min. Next the gel was immersed in solution B (3.5 mM silver nitrate) for 30 min. After rinsing with dH_2_O, a mixture of solution C (0.357 M) and solution D (4.37 mM) in dH_2_O were used to develop the protein spots on the 2D PAGE gel. The reaction was stopped by 5% acetic acid.

### 2.7. Detection and Quantitative Analysis of the 2D PAGE Gels

2D PAGE images were obtained, and the amount of protein in each spot was analyzed using ImageMaster 2D Elite software version 5.0 (Amersham Biosciences). The volume of a protein spot was defined as the sum of the intensities of the pixel units within the protein spot. To correct for quantitative variations in the intensity of protein spots, spot volumes were normalized as a percentage of the total volume of all the spots present in a given gel.

### 2.8. Protein Identification by LC-MS/MS

The process is described in a document from the Institute of Biological Chemistry, Academia Sinica Institute of Biological Chemistry (http://proteome.sinica.edu.tw/) and this was used with minor modification. Selected silver-stained protein spots were excised from the 2D PAGE. They were then de-silver-stain using 1 : 1 Na_2_S_2_O (0.1 g in 1 mL H_2_O) and K_6_Fe(CN)_6_ (0.1 g in 1 mL H_2_O) in 25 mM ammonium bicarbonate until being transparent. Next the gel slices were added to 25 mM ammonium bicarbonate pH 8.5 for 10 min, vacuum-dried, and rehydrated with 50% acetonitrile in 25 mM ammonium bicarbonate pH 8.5 at room temperature for 10 min. The supernatant was then discarded and the sample vacuum-dried. Subsequently, the protein within the spot was trypsinized with 0.1% sequencing-grade modified trypsin (Promega, Madison, WI, USA) in 25 mM ammonium bicarbonate, pH 8.5, at 37°C for at least 16 hr. Each supernatant was removed into a new Eppendorf tube, then 25 mM ammonium bicarbonate pH 8.5 was added to the previous gel sample, and the mixture sonicated for 1 min, 10 times. The supernatant from this treatment was also removed into a new Eppendorf tube. The process was repeated a third time by adding 50% acetonitrile in 25 mM ammonium bicarbonate pH 8.5 to the sample, which was followed by sonication for 1 min, 10 times. The three extracted supernatants were pooled and evaporated to dryness under vacuum, and the dried pellet used to carry out integrated nanoLC-MS/MS system (Micromass) (National Research Program for Genomic Medicine, Academia Sinica). Eleven rare differentially regulated protein spots were identified by LC-MS/MS (Orbitrap) mass spectrometry system (Proteomics Research Center, National Yang-Ming University) in this way. The nanoLC-MS/MS data acquisition was carried out by Micromass ProteinLynx Global Server (PGS) 2.0 data processing software in a default mode and outputted as a single Mascot-searchable peak list (.pkl) file. The LC-MS/MS (Orbitrap) data were processed by SWQUEST (Thermo Finnigan). The peak list files were used to query the Swiss-Port version 2010_05 database using Mascot program version 2.2 (release date, 28-Feb-2007, Matrix Science, London, UK) with the following parameters: a taxonomy of *Homo sapiens* (20,400 sequences), peptide mass tolerance of 50 ppm, MS/MS ion mass tolerance of 0.25 Da, trypsin digestion with one missed cleavage, no fixed modification, and the variable modifications considered were methionine oxidation, cysteine carboxyamidomethylation, lysine acetylation and phosphorylation of tyrosine, serine, and threonine. Only significant hits as defined by Mascot probability analysis were considered. Protein identifications were accepted with a statistically significant Mascot protein search score ≥36 or SEQUEST score = 2.5 (critical), which corresponds to an error probability of *P* < 0.05 using our data set. The protein identification with the highest score was selected to eliminate protein redundancy within the database.

### 2.9. Cluster Analysis and Functional Classification of the Differentially Expressed Proteins

A plot of the calibrated intensity for the expression value of each protein as measured by ImageMaster 2D Elite software version 5.0 (Amersham Biosciences, Sweden) among the different groups of samples was used in conjunction with an average linkage hierarchical clustering algorithm (UPGMA, Unweighted Pair Group Method with Arithmetic Mean); this was done using Hierarchical Clustering Explorer 3.5 [17]. The uncentered Pearson's correlation coefficient was determined as a measurement of the similarity metric, and the threshold value for the minimum similarity was set at 0.8. After clustering, each protein was allocated its place in a global temporal classification color heat map. We used BGSSJ (Bulk Gene Search System for Java; http://bgssj.sourceforge.net/) [17] and the Swiss-Prot protein knowledge database for the functional classification of the proteins.

### 2.10. Western Blotting

Proteins extracts from fibroblast cells (Detroit 551) were separated by 12.5% SDS-PAGE and then transferred onto a nitrocellulose (NC) membrane. The NC membrane was blocked with 5% nonfat milk in TBST at room temperature for 1 hr and probed with various primary antibodies (anti-p-Erk1/2 (no. 9101), 1 : 2000; anti-Erk1/2 (no. 9102), 1 : 1000; anti-p-Akt (no. 9271), 1 : 1000 and anti-Akt (no. 9272), 1 : 1000, Cell Signaling; anti-TGF-*β* (sc-52829), 1 : 1000, Santa Cruz; anti-*β*-tubulin (T4026), 1 : 5000, Sigma Aldrich; anti-HSPB1 (ab39399), 1 : 1000 and anti-STMN (cb1047), 1 : 1000, Abcam; anti-GSTP1 (GTX112953), 1 : 3000 and anti-TPIS (GTX104618), 1 : 3000, GeneTax) in 5% BSA or non-fat milk in TBST. After washing the NC membrane, the NC membrane was treated with HRP-conjugated secondary antibody. The bands were visualized by chemiluminescence using a chemiluminescence imaging system (LAS-4000, Fujifilm) and performed densitometric analysis using Fujifilm MultiGauge software ver. 2.0. The results are expressed as mean ± standard deviation. Student's *t*-test was used to evaluate statistical significance, and a *P* value <0.05 was regarded as statistical significant (*n* = 4 for each experiment).

### 2.11. Intracellular ROS (Reactive Oxygen Species) Assay

To measure the ROS content of the fibroblasts after DMSO, AS extract, or FA treatment, the intracellular content of H_2_O_2_, which is in proportion to ROS, was determined using the redox-sensitive fluorescent dye 2′,7′-dichlorofluorescein diacetate (DCF-DA) (Sigma). Briefly, cells were cultured to confluence and trypsinized. After centrifugation, discard supernatant and the cells were resuspended and incubated with 10 *μ*M DCF-DA (20 mM in DMSO for stock solution and stored in −20°C), which solved in 1 × PBS for 10 minutes at 37°C in the dark. Centrifuge again and cells were then washed and resuspended in fresh culture medium. A total of 8 × 10^4^ cells were added to a black flat 96-well ELISA plate with 200 *μ*L of medium. ROS is able to oxidize DCF-DA to the fluorescent DCF, and therefore the relative concentration of intracellular ROS is determined by a fluorescence reader using excitation at 485 nm and emission at 538 nm. Student's *t*-test was used to evaluate statistical significance, and a *P* value < 0.05 was regarded as statistical significant (*n* = 3 for each experiment).

### 2.12. Boyden Chamber Migration Assay

After cells were cultured to confluence, they were trypsinized, centrifuged, and resuspended in culture medium. A total of 2 × 10^4^ fibroblasts were seeded in a Transwell (24 well, Corning) and treated with DMSO, AS extract, or FA; the Transwell was then inserted in culture medium without bubbles. After 6 hr, the Transwell was removed from the culture medium and the cells fixed using methanol for 10 min. The methanol was then discarded and the cells air-dried. Finally the cells were stained with 5% Giemsa (solved in dH_2_O) in room temperature overnight. The Transwell was washed with dH_2_O, and the inner cells on the Transwell were removed by scraping with a cotton swab. The number of cells migrated down through the Transwell was manually counted under microscope. Student's *t*-test was used to evaluate statistical significances, and a *P* value <0.05 was regarded as statistical significant (*n* = 3 for each experiment).

### 2.13. Wound Healing Assay

A total of 2.5 × 10^4^ fibroblasts were seeded onto both sides of a culture insert (ibidi) in a 24-well plate to generate 500 *μ*m ± 50 *μ*m gap between the cell layers before drug treatment. After the cells had attached, the culture insert was removed carefully. The cells were then treated with the various drugs, which were added to the culture medium. The cells were then incubated for 24 hr. The medium was then discarded and the cells washed twice with PBS to remove unattached cells. Photographs were then taken by digital camera using microscopy (Olympus) and the size of the gap measured. Student's *t*-test was used to evaluate statistical significances, and a *P* value < 0.05 was regarded as statistical significant (*n* = 3 for each experiment).

### 2.14. Sircol Collagen Assay

The process is described in Sircol collagen assay general protocol (Biocolor). In brief, 3 mL cell medium was collected after drug treatment, and collagen was precipitated by 4 M NaCl to avoid FBS interference. The collagen pellet was collected by centrifugation at 15000 ×g in room temperature, and the pellet was dissolved in 0.5 mL of 0.5 M acetic acid. 1 mL of Sircol dye reagent was mixed with 100 *μ*L resolved collagen sample and then gently inverted at room temperature for 30 min. The resulting collagen was collected then centrifugation at 10000 ×g for 10 min. The pellet was dissolved in 1 mL alkali reagent by vortexing and the collagen concentration was determined by a spectrophotometer at OD540. Student's *t*-test was used to evaluate statistical significances, and a *P* value < 0.05 was regarded as statistical significant (*n* = 3 for each experiment).

## 3. Results

### 3.1. Safety and Efficacy Analysis of AS Ethanol Extracts and FA Treatment

**Figure 1:**
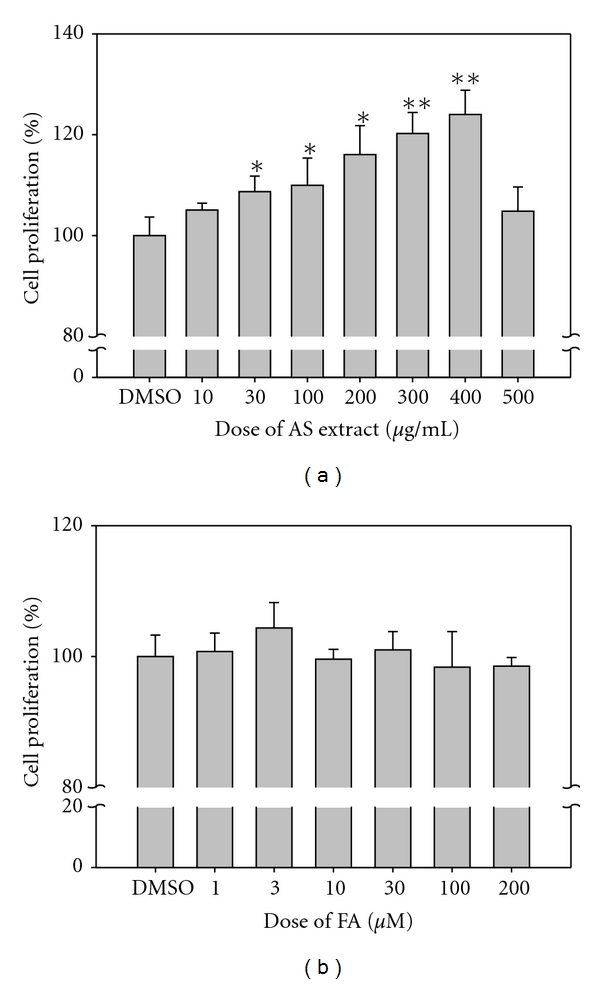
Effects of AS extract or FA on cell viability after 24 hr treatment in human fibroblast. Graph represents the ratio of cell viability compared with the DMSO control. (a) AS extract promotes cell growth in a dose dependent manner up to 400 *μ*g/mL, but FA treatment (b) has no significant effect on cell proliferation. Fibroblasts were seeded in a 24 well plate with 4 × 10^4^ cells per well. After the cells were attached, different drug concentrations were added for 24 hr and cell proliferation was evaluated by WST-1 assay at OD_450_−OD_690_. The control was treated with 0.1% DMSO only. The Student's *t* test was used to evaluate the statistical significance of the results, which are presented as mean ±SD (*n* = 5). *P < 0.05, **P < 0.01, compared with the control.

In order to establish a model system to analyze the efficacy of total herbal extracts, we used *Angelica sinensis* ethanol extracts (AS extract) and its active component, ferulic acid (FA), as samples. AS was extracted using absolute ethanol and then analyzed by HPLC to determine the quantity of FA in the AS extract. The concentration of FA in the AS extract (see Figure 1 in the supplementary material available at doi:10.1155/2012/467531) was 2.278 mg/mL. Traditionally, AS is a common basic component when formulating Chinese herbal medicines for wound healing, and therefore we used human skin fibroblasts as an experimental cell model to elucidate the underlying mechanisms by which AS aids with the process of wound healing. AS extract and FA were dissolved in DMSO to give concentrations ranging from 10 to 500 *μ*g/mL and 1 to 200 *μ*M, respectively, in order to determine the optimal drug concentrations for fibroblast cell growth. As shown in Figure 1, AS extracts were able to significantly enhance cell viability by up to 125% in a dose-dependent manner (Figure 1(a)), but FA had no obvious effect on cell growth (Figure 1(b)). It was noted that 500 *μ*g/mL of AS extract was the highest concentration that was still able to help cell growth, and concentration beyond this point inhibited cell growth. Based on these observations, we chose 300 *μ*g/mL of AS extract for this study in order avoid any complicated pharmacological effects. Correspondingly, the appropriate concentration of FA based on the level in the AS extract was 3.5 *μ*M. Noticeably, AS extract significantly promotes cell growth, but the active component FA is not able to induce a similar effect. This confirms that the pharmacological effect of a single component of an herb plant is not equivalent to that of a whole extract from the same plant in this case.

### 3.2. 2D PAGE Protein Profiling and the Identification of Differentially Expressed Protein Spots from Fibroblast Cells Treated with DMSO, AS Extract, or FA

**Figure 2:**
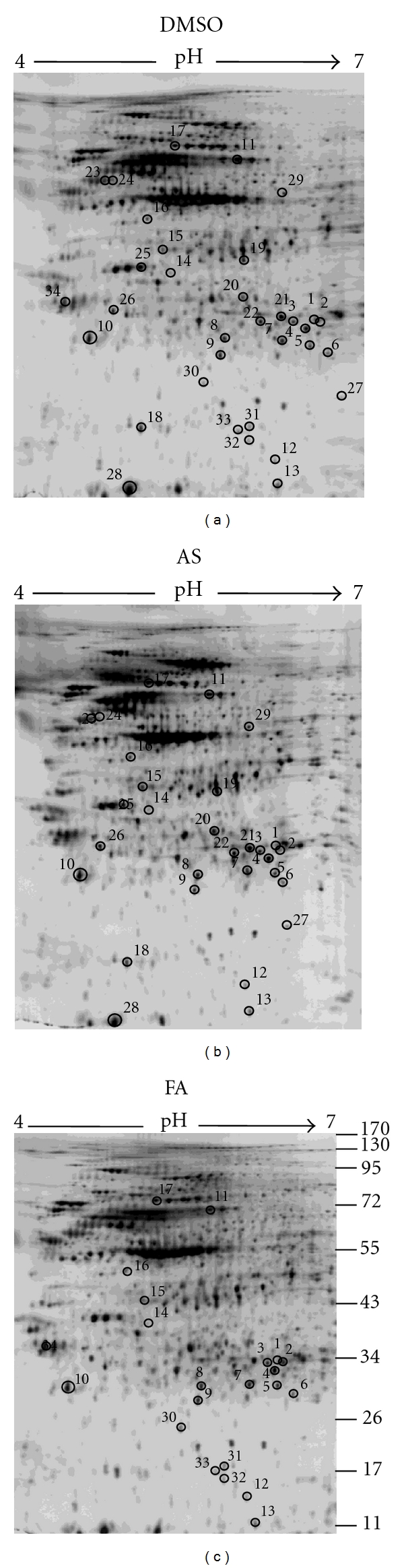
Differential effect of AS and FA treatment on human fibroblasts as visualized by 2D PAGE. 300 *μ*g/mL AS extract and 3.5 *μ*M FA were used to treat cells for 24 hr. Whole-cell lysates were extracted and analyzed by 2D PAGE (first dimension, 18 cm, pH 4–7, nonlinear gradient of IPG strips; second dimension, 12.5% SDS-PAGE) and visualized by silver staining. There were 29 and 22 differentially expressed spots that could be identified from the AS extract and FA-treated cells, respectively, compared with the DMSO control. The numbered protein spots were identified by LC-MS/MS mass spectrometry. The corresponding protein spot identities are shown in Table 1.

To further investigate how AS extract and its active component FA can affect the processes of wound healing of the injured skin, two-dimensional (2D) PAGE was then carried out. In this study, 0.1% DMSO, 300 *μ*g/mL AS extract, or 3.5 *μ*M FA-treated cell lysates were separated by 2D PAGE and visualized by silver staining. The stained 2D PAGE gels were then analyzed by Image Master 5.0. This software identified proteins showing quantitatively different expression levels on the 2D PAGE gels using the DMSO treated cells as reference. As shown in Figure 2, we were able to identify 29 and 22 protein spots for AS and FA respectively that were significantly increased or decreased by more than 1.5- or 0.67-fold in intensity in response to AS extract, or FA treatment compared to DMSO, respectively. These protein spots were numerically labeled and are shown in Figure 2.

 To further characterize these differentially expressed proteins, the concentration of total protein lysate was increased to more than 1 mg, which was followed by 2D PAGE and staining with Coomassie blue. Forty abundant differentially regulated protein spots were identifiable on this 2D PAGE gel, and these were excised and analyzed by LC-MS/MS (Q-TOF) mass spectrometry. In addition, eleven rare differentially regulated protein spots were excised from the earlier silver stained 2D PAGE gel and analyzed by the more sensitive LC-MS/MS (Orbitrap) mass spectrometry. The resulting peptides were screened using the Mascot database online search engine and SEQUEST to identify the proteins. The detailed results of the protein identification are presented in Table 1 using the DMSO treatment as reference. A Mascot score ≧36 or a SEQUEST score = 2.5 (critical) indicates significant protein homology (*P* < 0.05). As shown in Figure 2, 29 and 22 differentially expressed protein spots were characterized from the cell extracts treated with AS extract or FA, respectively.

### 3.3. Functional Classification of the Differentially Displayed Proteins

**Figure 3:**
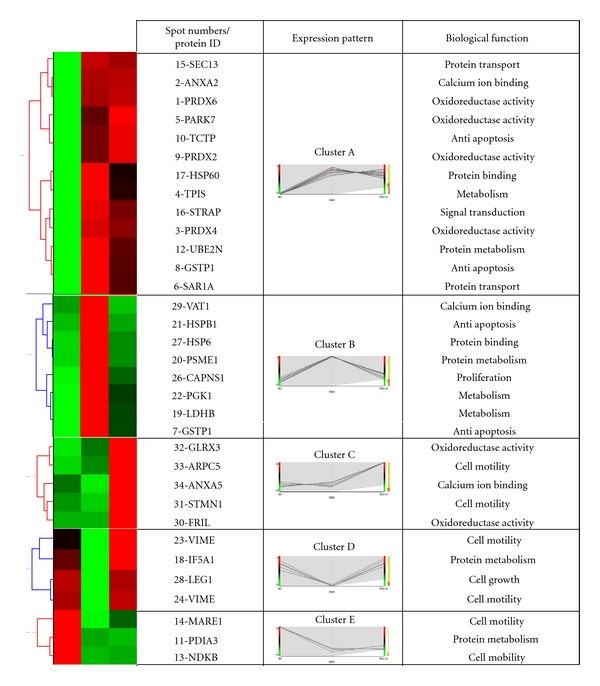
Hierarchical clustering and functional classification of the changes in protein expression between the DMSO control cells and AS/FA-treated cells. The expression pattern for each protein was categorized by UPGMA using Hierarchical Clustering Explorer 3.5. Proteins with a similar expression clustered into five different discrete groups (clusters A, B, C, D, and E) in a tree-like organization. Each row in the color heat map indicates a single protein and each column represents different groups of proteins from the DMSO, AS, and FA treatments. A high protein expression value for a specific protein spot is represented by a bright red color, and a low protein expression value is represented by a bright green color. A black color indicates that the protein spot was expressed at an average level. A gray color represents protein spots that were undetectable. Further information about the differentially expressed protein spots that were identified is given in Table 1. The functional classification of each protein was determined using BGSSJ and the Swiss-Prot protein sequence database.

Bioinformatic tools were used to categorize the differentially expressed proteins. All of the parameters of the proteins identified by mass spectrometry (Table 1), such as protein ID and ratio of protein expression levels, were introduced into HCE 3.5 (Hierarchical Clustering Explorer), which has been widely used for genomic or proteomic data mining and pinpointing hidden meaningful results [17, 18]. Based on the expression level ratios of the protein spots after the various treatments (AS extract/DMSO; FA/DMSO, Table 1), it was possible to categorized 34 protein spots into five clusters (Figure 3). In cluster A, the 13 protein spots are all upregulated in fibroblasts on treatment with either AS extract or FA. In cluster B, the 8 protein spots are upregulated in fibroblasts treated with AS extract, but the expression levels of these protein spots are not significantly changed upon treatment with FA. Five protein spots make up cluster C, and these are only overexpressed in fibroblasts treated with FA. In cluster D, 5 protein spots are downregulated when the fibroblasts was treated with AS extract, but there is no change in the expression level on treatment with FA. Only 3 protein spots form cluster E, and these are downregulated upon treatment with either AS extract or FA. It is worth noting that, except in clusters A and E, the protein expression is different when cells are treated with either AS extract or FA.

After clustering, we use NCBI PubMed and BGSSJ (Bulk Gene Search System for Java) with the Swiss-Port database to classify the biological functions of these proteins. The delineated protein functions included protein transport, protein metabolism, calcium ion binding, oxidoreductase activity, antiapoptosis, protein binding, metabolism, signal transduction, cell mobility, and cell growth.

 Interestingly, seven oxidation-related proteins were found among these differentially expressed proteins. Among them, five oxidation-related proteins were found to be upregulated in fibroblasts when treated with either AS extract or FA. These proteins were peroxiredoxin (PRDX) 2, PRDX4, PRDX6, glutathione S-transferase Pi (GSTP1), and Parkinson's disease protein 7 (PARK7) (Figure 3, cluster A). The other two oxidation related proteins, glutaredoxin-3 (GLRX3) and ferritin light chain (FRIL), were only up regulated in cells treated with FA (Figure 3, cluster C). The protein expression patterns of these proteins imply that FA alone might exert greater antioxidative ability on the fibroblasts than AS extract. Furthermore, four of the identified proteins were involved in the regulation of cell growth or antiapoptosis, namely, translationally controlled tumor protein (TCTP), heat shock protein beta-1 (HSPB1), GSTP1 (bifunctional protein), and calpain small subunit 1 (CAPNS1) (Figure 3, clusters A and B). TCTP was upregulated in fibroblasts treated with either AS extract or FA (Figure 3, cluster A). In contrast, HSPB1, GSTP1, and CAPNS1 were upregulated only in cells treated with AS extract (Figure 3, cluster B). This suggests that AS extract has a greater effect on cell survival compared to FA. These bioinformatic findings are consistent with the results from the WST-1 cell growth test (Figure 1).

Calcium ions are very important in the regulation of wound healing process, and its function is to modulate the expression of genes involved in cell growth, differentiation, attachment, motility, and collagen secretion [19]. Some proteins related to the above functions were identified in this study. Expression of annexin A2 (ANXA2) was upregulated in fibroblasts when treated with either AS extract or FA (Figure 3, cluster A); however, annexin A5 (ANXA5) was only upregulated when the cells were treated with FA (Figure 3, cluster C). In contrast, synaptic vesicle membrane protein VAT-1 homolog (VAT1) was only upregulated in cells treated with AS extract (Figure 3, cluster B), while triosephosphate isomerase (TPIS) was upregulated in cells treated with either AS extract or FA. Furthermore, phosphoglycerate kinase 1 (PGK1) and L-lactate dehydrogenase B chain (LDHB) were only upregulated in cells treated with AS extract. During the wound healing process, cells are hypoxic [20] and cell proliferation consumes a lot of energy in order to repair the injured tissue. TPIS and PGK1 are important to glycolysis, and LDHB catalyzes the generation of lactate by the reduction of pyruvate. Overexpression of these proteins would seem to provide additional energy resource allowing the cells to survive under stress.

A number of motility-associated proteins were also identified. Actin-related protein 2/3 complex subunit 5 (ARPC5) and stathmin (STMN1) were only upregulated in cells treated with FA (Figure 3, cluster C), while vimentin (VIME) was only downregulated in cells treated with AS extract (Figure 3, cluster D). Surprisingly, NDKB and MARE1 were down-regulated in cells after both treatments (Figure 3, cluster E). These results suggest that treatment of fibroblasts with either AS extract or FA improves the ability of the cell to survive under environmental stresses. With the exception of VIME (for mobility), treatment of fibroblasts with AS extract modulates the upregulation of proteins that are associated with cell growth (LEG1, CAPNS1), metabolism (PGK1, LDHB), calcium ion regulation (VAT1), and antiapoptosis (HSPB1). On the other hand, FA may have the unique ability (Figure 3, cluster C) to modulate the upregulation of proteins associated with cell mobility (STMN1, ARPC5), antioxidative functions (GLRX3, FRIL), and calcium ion regulation (ANXA5). Interestingly, we found some overlapping of proteins induced by either AS extract or FA treatment. Two proteins associated with cell mobility (MARE1, NDKB, Figure 3, cluster E) are down-regulated when fibroblasts are treated with either AS extract or FA. In contrast, some proteins associated with metabolism (TPIS), antioxidative functions (PRDXs, PARK7), antiapoptosis (GSTP1, TCTP), and calcium ion regulation (ANXA2) are upregulated when fibroblasts are treated with either AS extract or FA (Figure 3, cluster A).

### 3.4. Confirmation of the Differential Expression of the Various Proteins Detected by Proteomics Analysis

**Figure 4:**
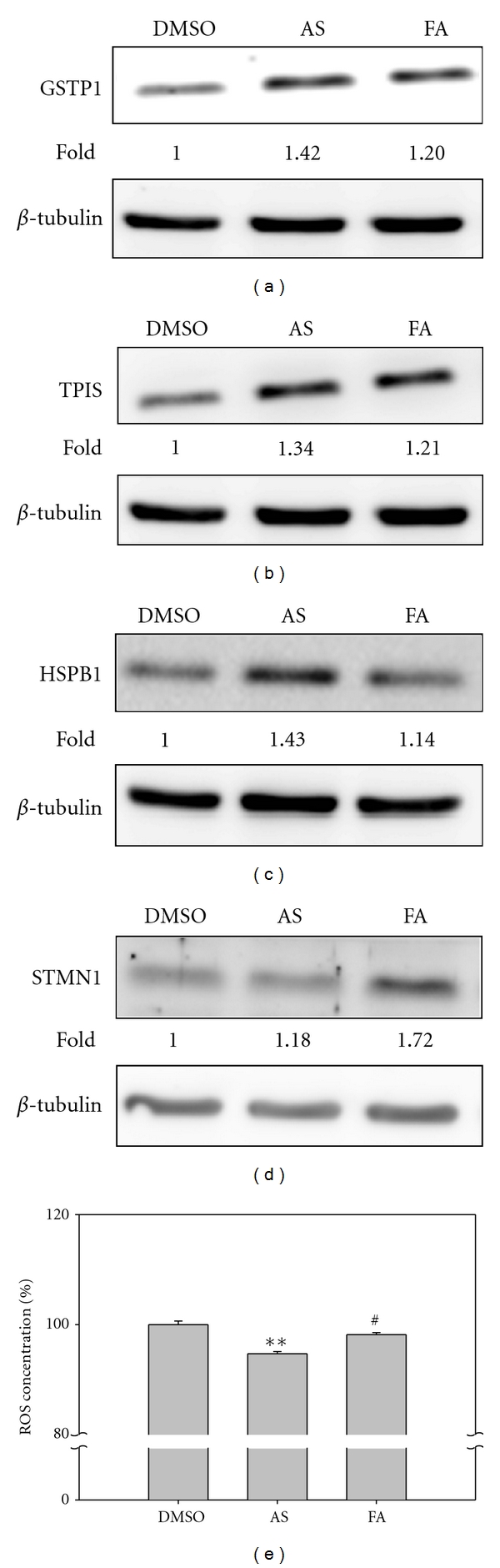
Analysis of the results of 2D PAGE by immunoblotting and ROS assay. According to hierarchical clustering and functional classification, the expressions of GSTP1 and TPIS were both increased in 300 *μ*g/mL AS extract and 3.5 *μ*M FA-treated cells. In addition, STMN expression increased in AS-extract-treated cells, and HSPB1 expression increased in FA treated cells. GSTP1 expression was examined by immunoblotting (a) using anti-GSTP1 antibody and these results were then further confirmed by functional ROS assay (e). The expression of TPIS (b), HSBP1 (c), and STMN (d) were also confirmed by immunoblotting using specific antibodies. In all cases the results correlated with the clustering and functional classification of 2D PAGE. *β*-Tubulin was employed as the sample loading control. The relative expression levels were detected by Fujifilm Multigauge ver. 2.0 for densitometric analysis and normalized with internal control, and a Student's *t* test was performed to evaluate the statistical significance. Results are presented as mean ± SD (*n* = 3). *P < 0.01, ^#^ *P* < 0.1 but with a decreased trend compared with DMSO.

Western blot analysis was used to confirm the changes in protein expression detected by the proteomic approach. Human skin fibroblasts were seeded into plates and then treated with either AS extract or FA for 24 hr; then cell lysates were prepared as indicated in Materials and Methods. As shown in Figures 4(a) and 4(b), expression of GSTP1 and TPIS (Figure 3, cluster A) were upregulated in fibroblasts after treatment with either AS extract or FA, which is consistent with our proteomic analysis (Figure 3 and Table 1). HSBP1 (Figure 3, cluster B) was significantly upregulated in fibroblasts after AS extract treatment, but FA treatment did not significantly change expression of this gene (Figure 4(c)). Furthermore, STMN1 was only significantly upregulated in cells treated with FA and no such change was detected when the cells were treated with AS (Figure 4(d)); this also agrees with the proteomic analysis (Figure 3, cluster C). Thus western blot analysis of selected proteins is in agreement with the expression pattern of the proteins as detected by our 2D PAGE and mass spectrometry analysis (Figure 3), which provides validation of our approaches.

### 3.5. Redox State of the Fibroblast after Treatment with AS Extract and FA

The PRDX family of proteins and GSTP1 act as antioxidants [21], and these proteins show the greatest increase in expression in response to treatment with AS extract or FA (Table 1). These results seem to indicate that the increase in these proteins may help to change the redox state of the cells after drug treatment. The redox-sensitive fluorescent dye 2′,7′-dichlorofluorescein diacetate (DCF-DA) was used to measure the redox state of cells after drug treatment. As shown in Figure 4(e), the ROS level of cells treated with AS extract was significantly reduced by 8%, but this did not occur when the cells were treated with FA. It is worth noting that FA is an effective ROS scavenger due to its structure [11]; nonetheless, it would seem that FA alone is not completely effective in terms of antioxidant activity. Thus some component(s) from the AS extract may be necessary, together with FA, to obtain maximum anti-oxidative activity.

### 3.6. Functional Confirmation of the Effect of AS Extract on Collagen Secretion and Migration

**Figure 5:**
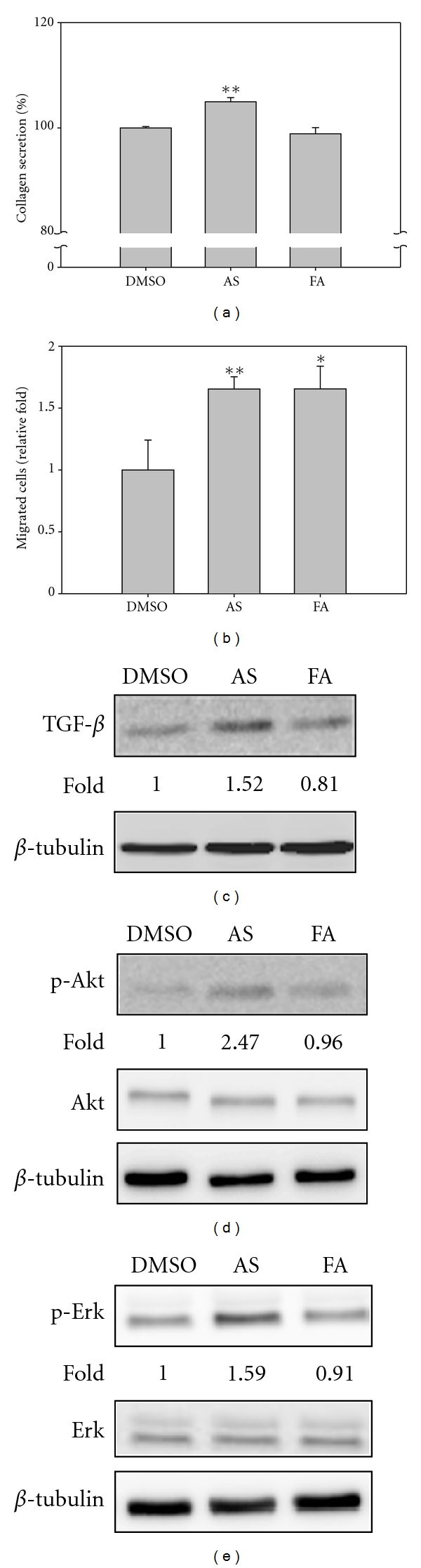
Functional confirmation of the effects of AS and FA treatment on human fibroblasts. (a) Collagen secretion was significantly increased after AS extract treatment. After cells were seeded in a 24 well plate and treated with 0.1% DMSO, 300 *μ*g/mL AS extract or 3.5 *μ*M FA for 24 hr, the medium were collected to analyze the collagen concentration. The graph represents the ratio of collagen secretion compared to the DMSO control. (b) Fibroblast migration is promoted after treatment with AS extract or FA. A total of 2 × 10^4^ cells were seeded into the top of Boyden chamber with 0.1% DMSO, 300 *μ*g/mL AS extract or 3.5 *μ*M FA and added fresh culture medium into the bottom. After 6 hr, the cells were stained by 5% Giemsa dissolved in water and the upper cells on Boyden chamber were scraped with cotton swabs then the cells counted using an inverted microscope. (c) TGF-*β* expression was increased after AS extract treatment and was correlated with collagen secretion (a). A total of 1 × 10^6^ fibroblasts were seeded onto a 10 cm culture dish and then treated with DMSO, 300 *μ*g/mL AS extract or 3.5 *μ*M FA for 24 hr. After washing with 1 × PBS, the cells were lysed by NP-40 lysis buffer with protease and phosphatase inhibitor. TGF-*β* expression was determined by immunoblotting. The expression of phospho-Akt (d) and phospho-Erk (e) were also examined by immunoblotting and it was found that AS extract was able to significantly increase expression of these proteins. The relative expression levels were detected by Fujifilm Multigauge software and normalized with *β*-tubulin. The results are expressed as mean ± SD (*n* = 3). *P < 0.05, **P < 0.01, compared with the control.

Collagen secretion and crosslinking are the most important step when repairing wounded tissue and collagen synthesis is controlled by transforming growth factor beta (TGF-*β*), which is secreted by macrophages and fibroblasts [22]. Thus the effect of AS extract and FA on the secretion of collagen by fibroblasts was examined. As shown in Figure 5(a), AS extract was able to significantly promote collagen secretion by fibroblasts and the increase was up to 5% compared to the control; in contrast, FA did not increase collagen secretion. Expression of TGF-*β* was also analyzed by Western blotting, and it was found that expression of TGF-*β* in response to treatment with either AS extract or FA correlated with the results of the collagen secretion assay (Figure 5(c)).

The functions of STMN1, ARPC5, NDKB, and MARE1 are known to be associated with cell mobility and wound healing [23–26]; however, the expression levels of these proteins, as detected by our proteomic analysis, varied, and they grouped into different clusters (Figure 3, cluster C, D, E) in response to drug treatment. In these circumstances it is important to clarify the effects of each treatment on the fibroblasts, and therefore cell migration and wound healing assays were carried out on cells treated with either AS extract or FA. The Transwell assay was used for the cell migration test. As shown in Figure 5(b), we found that both AS extract and FA seemed to promote cell migration to the same degree. Furthermore, both AS extract and FA had the same effect on the promotion of fibroblast wound healing (supplementary Figure 2). These results suggest that FA might be the major active component in AS extract that promotes cell migration and wound healing.

Only CAPNS1 is involved in cell growth based on the proteomic classification (Figure 3, cluster B). Phosphorylated Akt and Erk are known to be the factors that are involved in promoting cell growth, and therefore experiments were performed to measure the expression of these two factors in fibroblasts after treatment with either AS extract or FA. As shown in Figures 5(d) and 5(e), the expression levels of p-Akt and p-Erk were only upregulated in fibroblasts treated with AS extract. These results are consistent with our cell viability assay, which shows that fibroblast cell growth is increased by about 25% when fibroblasts are treated with AS extracts, but this did not occur when the cells are treated with FA (Figure 1). These findings indicate that cell growth promotion is initiated by AS extract treatment and this is likely to involve upregulation of the Akt and Erk pathway in the treated fibroblast cell.

## 4. Discussion

The cell viability test revealed that AS extract was able to effectively promote human skin fibroblast proliferation with low levels of cytotoxicity even at high concentrations. It is well known that skin fibroblasts play an important role in the proliferation phase of wound healing [27, 28], and therefore treatment with AS extract would seem to have beneficial effects on fibroblast growth. It has been found previously that functionally related proteins are often coordinately expressed [29]. In agreement with this hypothesis, five clusters of proteins were identified using 2D PAGE analysis with similar changes in cellular expression level in terms of AS/FA treatments.

### 4.1. Proteins Involved in Antioxidative Activity

ROS plays an important role in the early phase of wound healing [30], but excessive ROS in the wounded region is also harmful to nearby normal cells. Peroxiredoxins (PRDXs), Parkinson's disease protein 7 (PARK7), and glutathione S-transferase Pi (GSTP1) were categorized as cluster A proteins that are upregulated in fibroblasts by either AS extract or FA (Figure 3, cluster A). PRDXs are a family of multifunctional antioxidant thioredoxin-dependent peroxidases that protect cells from hydrogen peroxide [31, 32]. The PRDXs family consists of six members, and three of these (PRDx2, PRDX4, and PRDX6) were identified as upregulated in this study. PARK7 acts as a coordinator and induces the expression of antioxidant genes such as glutamate cysteine ligase and glutathione [33]. GSTP1 is a multifunctional protein that can reduce hydroperoxides, carry out detoxification, and inhibit JNK phosphorylation; the latter results in a decrease apoptotic cell numbers [34]. The mass spectrometry analysis indicated that PRDX-2, PDRX-4, PDRX-6, and PARK7 were identified in our sample (Table 1) with lower sequence coverage. Nevertheless, the amount of hydrogen peroxide in the fibroblasts after AS extract treatment was substantially reduced (Figure 4(e)), which indicates the presence of antioxidants in cells after drug treatment. Furthermore, GSTP1 was significantly upregulated in fibroblasts that had been treated with either AS extract or FA (Figure 4(a)). Together our results reveal that ROS was indeed significantly decreased in cells after AS extract or FA treatment and that it is likely that PRDX-2, PDRX-4, PDRX-6, PARK7, and GSTP1 are involved in this process.

### 4.2. Proteins Involved in the Regulation of Apoptosis

TCTP (a translationally controlled tumor protein also named fortilin) and GSTP1 are upregulated by treatment with either AS extract or FA (Figure 3, cluster A); additionally, AS extract but not FA is able to induce heat shock protein beta 1 (HSPB1) expression (Figure 3, cluster B; Figure 4(c)). The N-terminus of TCTP is able to interact with Bcl-xL and help the cell to avoid apoptosis; in contrast, downregulation of TCTP expression increases the number of apoptotic cells [35, 36]. It has also been reported that down-regulation of GSTP1 results in an increase of c-Jun NH2-terminal kinase-mediated apoptosis [37, 38]. HSPB1 (HSP27) works as chaperone and helps protein folding; alternatively, it can activate the proteasome allowing it to interact with denatured proteins [39]. Phosphorylated HSPB1 is able to inhibit the interaction of Daxx with Fas or ASK1, which decreases the activation of downstream apoptosis-related proteins [40]. Furthermore, wound closure is delayed in knock-down SW480 cells indicating the involvement of HSPB1 in regulating cell mobility [41]. Our results seem to demonstrate that AS extract and FA may activate anti-apoptosis in cells by upregulating the expression of TCTP and GSTP1. Moreover, AS extract but not FA may have the additional function of inhibiting apoptosis and regulating cell mobility during the wound healing process via upregulation of HSPB1.

### 4.3. Proteins Involved in the Regulation of Calcium Ion or Protein Transportation

Calcium ions play an important role in wound healing, cell proliferation, differentiation, adhesion, migration, and collagen secretion [19]. Annexins are membrane proteins that contain the annexin core domain and a conserved calcium-lipid binding module that is involved in calcium ion regulation. In addition, expression of annexin A2 has been observed to be increased in migrating intestinal epithelial cells [42]. In this study it was found that annexin A2 was upregulated in fibroblasts treated with either AS extract or FA (Figure 3, cluster A). Moreover, a synaptic vesicle membrane protein VAT-1 homolog (VAT-1) also is known to participate in regulating calcium ion flux [43]. We found that treatment with AS extract but not with FA was able to induce the upregulation of VAT-1 (Figure 3, cluster B). Interestingly, our biochemical assays further indicated that collagen secretion as well as the expression of TGF-*β* is substantially increased in fibroblasts after AS extract treatment but not FA treatment (Figures 5(a) and 5(e)). Therefore we propose that AS extract has a greater capacity than FA to affect calcium ion regulation as well as any related wound recovery effects.

### 4.4. Proteins Involved in Cell Mobility

Cell mobility is an important part of the wound healing process [28]. Nucleoside diphosphate kinases B (NDKB) and microtubule-associated protein RP/EB family member 1 (MARE1) were both down-regulated in cells treated with either AS extract or FA (Figure 3, cluster E). NDKB (also named nm23) seems to be a metastasis suppressor gene and is thought to be involved in cell migration, proliferation, differentiation, and development [25]. MARE1 is also thought to promote polymerization of microtubule and to stabilize microfilaments [26]. Downregulation of NDKB and MARE1 in fibroblasts that are treated with either AS extract or FA would suggest that there is a reduction in the suppression of migration, which might be beneficial to cells involved in the wound healing process. Actin-related protein 2/3 complex subunit 5 (ARPC5) and stathmin (STMN1) were both upregulated by FA but not by AS extract (Figure 3, cluster C). ARPCs are able to regulate actin dynamics and function as a template for initiation of new actin filaments that branch off an existing filament [24]. STMN1 is cytosolic phosphoprotein and is known to act as microtubule destabilizing protein that binds free form tubulin, which inhibit microtubule polymerization [23]. Thus, our results indicate that both AS extract and FA are able to regulate cell mobility probably by controlling microtubule dynamics. Furthermore, it is worth noting that ARPC5, STMN1, and NDKB have the highest sequence coverage and score in our mass spectrometric analysis (Table 1). The migration and wound healing assays (Figure 5(b)) show that AS extract and FA both promote cell mobility, and this agrees with the above findings.

### 4.5. Proteins Involved in Cell Growth or Metabolism

CAPNS1 (calpain small subunit 1) is able to promote cell proliferation, migration, and adhesion [44]. In contrast, LEG1 (galectin-1) overexpression is thought to inhibit normal mouse fibroblast growth and increase apoptosis [45]. Interestingly, we observed that CAPNS1 was upregulated (Figure 3, cluster B), while LEG1 was down-regulated (Figure 3, cluster D) when fibroblasts were treated with AS extract; such contrasting changes in expression between CAPNS1 and LEG1 should be beneficial to the fibroblast wound healing process. Furthermore, our results also show that, when fibroblasts were treated with AS extract there were activations of p-Akt and p-Erk expression (Figures 5(d) and 5(e)). Thus, in addition to regulating the expression of CAPN1 and LEG1, AS extract seems to also modulate cell growth via the Erk and Akt pathways.

 The main energy resource of fibroblasts is glycolysis, and an increase in LDHB expression in these cells would promote propyl hydroxylase activity, which in turn would increase lactate production and collagen hydroxylation; these effects, in turn, would help cells tolerate an hypoxia environment and accelerate the wound healing process [46]. We found that both AS extract and FA are able to upregulate the expression of Triosephosphate isomerase (TPIS) in fibroblasts (Figure 3, cluster A), while only AS extract could activate the expression of phosphoglycerate kinase (PGK1) and L-lactate dehydrogenase B chain (LDHB) (Figure 3, cluster B). Thus we conclude that AS extract might have greater efficacy in providing extra energy for wound healing processes by upregulating glycolysis, which would help to protect cells from hypoxia.

### 4.6. Potential Molecular Pharmaceutical Mechanism of AS Extract and FA in Regulating Wound Healing Processes of Fibroblasts

**Figure 6:**
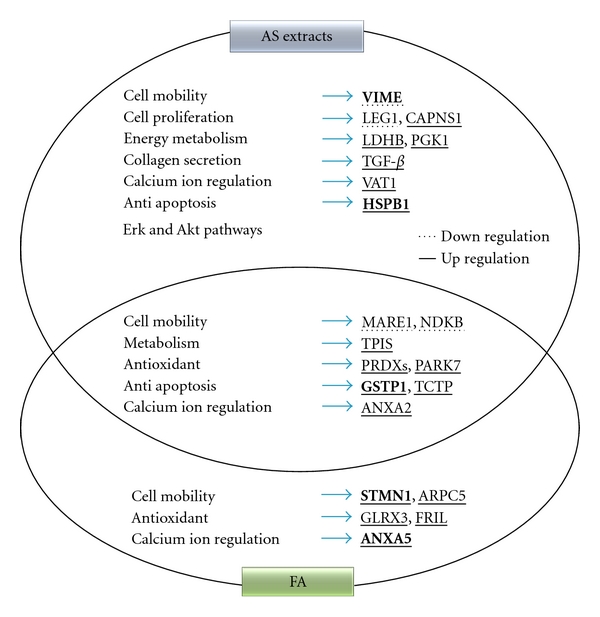
Summary of the effects of the AS and FA treatments on human skin fibroblasts.

Taken together, our results revealed a possible pharmacological wound healing processes induced by the AS extract or FA treatment of fibroblasts (Figure 6). Multiple factors are likely to be involved in the wound healing processes induced by either AS extract or FA. Both AS extract and FA seem to activate the inhibition of ROS production (PRDXs and PARK7), enhance cell mobility (MARE1 and NDKB), promote glycolysis (TPIS), increase antiapoptosis (GSTP1 and TCTP), and regulate calcium ion (ANXA2), all of which ought to increase the viability of cells during the wound healing process. The unique pharmacological functions of AS extract that have been revealed in this study include enhanced human skin fibroblast proliferation due to suppression of LEG1 expression, and increased CAPNS1 expression, and upregulation of PGK1 and LDHB, which promote glycolysis and activation of the Erk and Akt signal transduction pathways. Fibroblasts treated with AS extract seem also to undergo a suppression of apoptosis due to increased HSPB1 expression. Moreover, an increase in collagen secretion (TGF-*β*), calcium ion regulation (VAT1) and enhanced cell mobility (down-regulation of VIME) also contribute towards a more efficient wound healing process. In addition, a number of unique functions of FA have also been revealed, which include ROS inhibition (GLRX3 and FRIL) and the promotion of cell mobility by upregulation of STMN1 and ARPCS and down-regulation of NDKB. The extra functions of FA mentioned above should reinforce the healing process associated with fibroblasts as a whole. In this work, we have described a technological platform that is able to reveal the underlying principles of a herbal medicine; namely, how an AS extract and its active component FA participate in the modulation of wound healing process associated with fibroblasts. These results will help and benefit the clinical applications of this herbal medicine when it is used to treat heat burns or traumatic injury to the skin.

## Supplementary Material

Figure 1: HPLC analysis of AS ethanol extract and FA.
Figure 2: Wound healing assay of 0.1% DMSO, 300 **μ**g/ml AS ethanol extract and 3.5 **μ**M FA.

## Figures and Tables

**Table 1:** Proteins showing altered abundance after treatment with either AS extract or FA compared to the DMSO control and identified by LC-MS/MS.

ID	Protein identity	Accession number	Abbreviation	MW/pI	Score	Match peptide	Sequence coverage (%)		AS/DMSO		FA/DMSO
1	Peroxiredoxin-6	P30041	PRDX6	25019/6.00	40	3	12	↑	1.91	↑	1.96
2	Annexin A2	P07355	ANXA2	38580/7.57	110	3	9	↑	1.50	↑	1.51
3	Peroxiredoxin-4	Q13162	PRDX4	30521/5.86	105	4	14	↑	2.02	↑	1.88
4	Triosephosphate isomerase	P60174	TPIS	26713/6.45	236	7	27	↑	1.86	↑	1.52
5	Parkinson disease protein 7	Q99497	PARK7	19878/6.33	67	2	12	↑	1.57	↑	1.77
6	GTP-binding protein SAR1a	Q9NR31	SAR1A	22353/6.21	45	1	5	↑	1.72	↑	1.51
7	Glutathione S-transferase P	P09211	GSTP1	23341/5.43	214	7	43	↑	1.70	↑	1.50
8	Glutathione S-transferase P	P09211	GSTP1	23341/5.44	174	6	38	↑	2.54	↑	1.50
9	Peroxiredoxin-2	P32119	PRDX2	21878/5.66	135	5	23	↑	1.77	↑	1.96
10	Translationally controlled tumor protein	P13693	TCTP	195834.84	119	4	17	↑	1.57	↑	1.71
11	Protein disulfide-isomerase A3	P30101	PDIA3	56747/5.98	1019	27	40	↓	0.53	↓	0.51
12	Ubiquitin-conjugating enzyme E2	P61088	UBE2N	17127/6.13	2.5 (criteria)	4	26.3	↑	2.04	↑	1.77
13	Nucleoside diphosphate kinase B	P22392	NDKB	17287/8.52	70	4	28	↓	0.63	↓	0.63
14	Microtubule-associated protein RP/EB family member1	Q15691	MARE1	29980/5.02	117	4	20	↓	0.49	↓	0.62
15	Protein SEC13 homolog	P55735	SEC13	35518/5.22	130	4	9	↑	1.64	↑	1.60
16	Serine-threonine kinase receptor-associated protein	Q9y3f4	STRAP	38414/4.98	2.5 (criteria)	5	18.9	↑	1.97	↑	1.78
17	Heat shock protein 60	P10809	HSP60	61016/5.70	379	15	19	↑	1.99	↑	1.56
18	Isoform A of eukaryotic translation initiation factor 5A-1	P63241-2	IF5A1	20157/5.07	2.5 (criteria)	4	21.2	↓	0.56		
19	L-Lactate dehydrogenase B chain	P07195	LDHB	36615/5.17	115	5	17	↑	2.01		
20	proteosome activator complex subunit 1	Q06323	PSME1	28705/5.78	289	7	27	↑	2.77		
21	Heat shock protein beta-1	P04792	HSPB1	22768/5.98	223	9	37	↑	1.65		
22	Phosphoglycerate kinase 1	P00558	PGK1	44586/8.30	94	1	4	↑	2.05		
23	Vimentin	P08670	VIME	53619/5.06	2.5 (criteria)	10	23.8	↓	0.64		
24	Vimentin	P08670	VIME	53619/5.07	2.5 (criteria)	10	23.8	↓	0.43		
25	Annexin A5	P08758	ANXA5	35914/4.94	272	13	40	↓	0.47		
26	Calpain small subunit 1	P04632	CAPNS1	28298/5.05	2.5 (criteria)	8	36.2	↑	1.62		
27	Heat shock protein 6	O14558	HSP6	17125/5.95	75	4	38	↑	1.67		
28	Galectin-1	P09382	LEG1	14706/5.34	81	3	19	↓	0.66		
29	Synaptic vesicle membrane protein VAT-1 homolog	Q99536	VAT1	41893/5.88	2.5 (criteria)	8	28	↑	1.78		
30	Ferritin light chain	P02792	FRIL	20007/5.51	2.5 (criteria)	3	20			↑	2.48
31	Stathmin	P16949	P08758	17292/5.76	134	6	36			↑	1.96
32	Glutaredoxin	O76003	GLRX3	37408/5.31	152	5	18			↑	1.66
33	Actin-related protein 2/3 complex subunit 5	O15511	ARPC5	16310/5.47	179	6	27			↑	1.53
34	Annexin A5	P08758	ANXA5	35914/4.94	2.5 (criteria)	16	65			↑	1.53
