# Supplementary figures and images for: A Study of the Wound Healing Mechanism of a Traditional Chinese Medicine, Angelica sinensis, Using a Proteomic Approach
Source: Evid Based Complement Alternat Med. 2012 Mar 25;2012:467531. doi: 10.1155/2012/467531 (PMC3319019; doi:10.1155/2012/467531)

Supplementary Fig.1

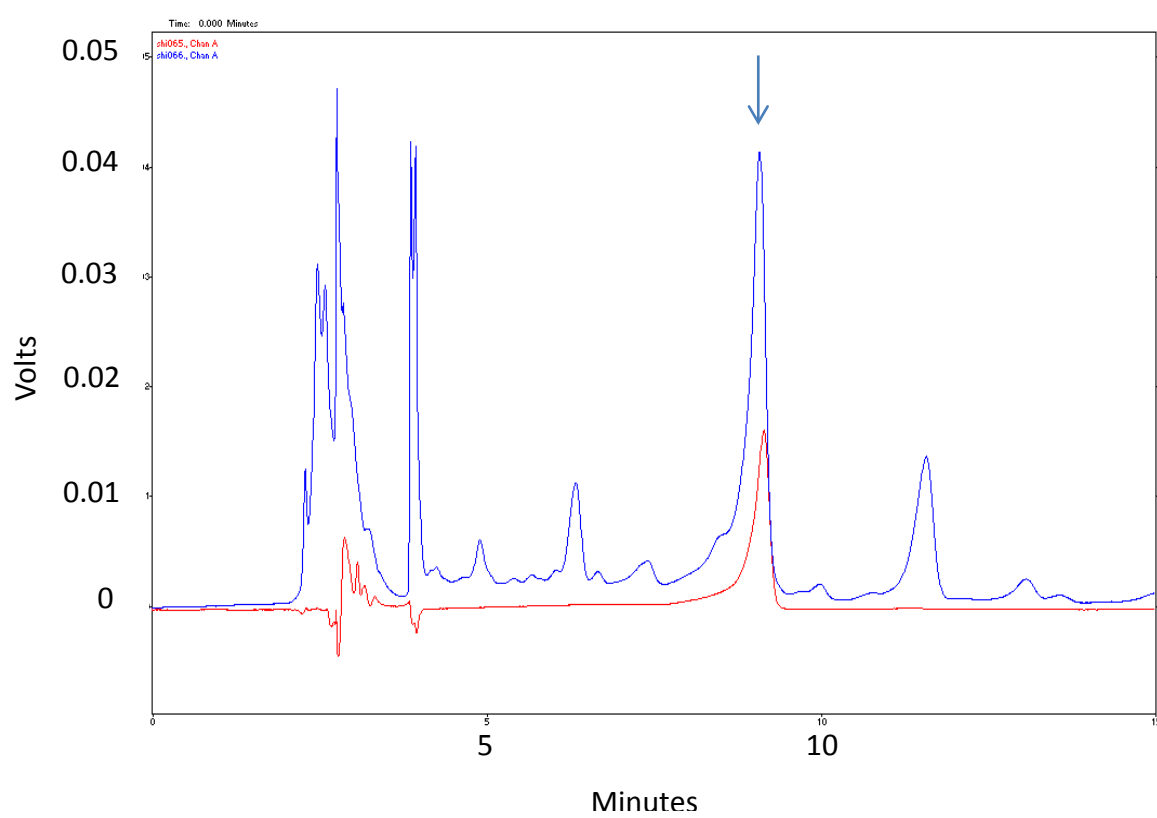

Fig.2

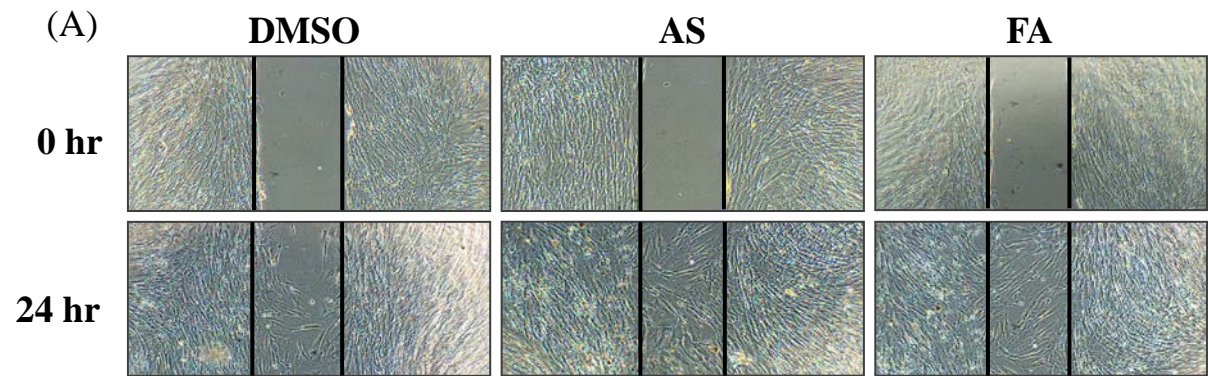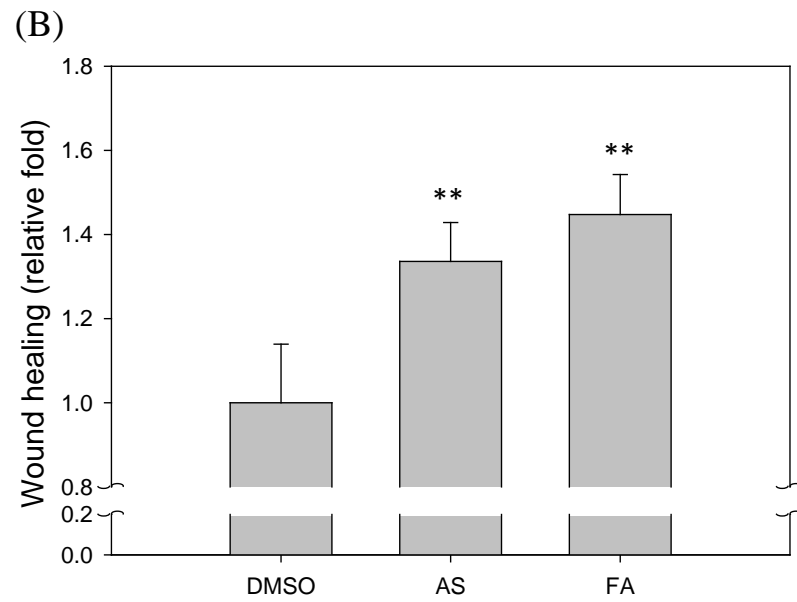

Supplement: Supplementary file 1 — Figure 1: HPLC analysis of AS ethanol extract and FA. Figure 2: Wound healing assay of 0.1% DMSO, 300 μg/ml AS ethanol extract and 3.5 μM FA. [file 467531.f1.pdf]
